# Cardiorespiratory Fitness and Performance in Multiple Domains of Executive Functions in School–Aged Adolescents

**DOI:** 10.3389/fphys.2021.640765

**Published:** 2021-03-02

**Authors:** Ludmila Lucena Pereira Cabral, Rodrigo Alberto Vieira Browne, Yuri Alberto Freire, Daniel Schwade, Gabriel Costa Souto, Matheus Dantas, Flávio Anselmo Silva Lima, Luiz Fernando Farias-Junior, Eduardo Caldas Costa, Jônatas França Barros

**Affiliations:** ^1^Graduate Program in Physical Education, Federal University of Rio Grande do Norte, Natal, Brazil; ^2^Graduate Program in Health Sciences, Federal University of Rio Grande do Norte, Natal, Brazil; ^3^Department of Physical Education, Federal University of Rio Grande do Norte, Natal, Brazil

**Keywords:** physical fitness, aerobic fitness, cognition, cognitive processing, adolescence, young

## Abstract

The objective of this study was to investigate the association between cardiorespiratory fitness (CRF) and performance in multiple domains of executive functions in school–aged adolescents. A sample of 132 adolescents (43% girls) aged 11–16 years were included in this cross–sectional study. Each participant completed a progressive aerobic cardiovascular endurance running (PACER) test, computerized cognitive tasks (Attentional Network, Berg’s Card Sorting, Go/No–Go oddball, Sternberg’s Working Memory, and Tower of London) and questionnaire for daytime sleepiness, as well as other factors that might influence cognitive performance (age, sex, school year, pubertal stage, and body mass index – BMI). Generalized linear model was used to calculate the coefficient estimates (β) and its 95% confidence interval (CI) for the cognitive tasks using PACER laps as a predictor, controlling for potential confounding variables. There was a negatively association of PACER laps with planning (β = –17.1, 95% CI –31.9, –2.3) and solution (β = –44.6, 95% CI –75.1, –14.2) time in performing the Tower of London task, as well as with perseverative errors in performing Berg’s task (β = –0.073, 95% CI –0.133, –0.013). Moderating effect of sex was found for the association of PACER laps with completed categories and perseverative errors in Berg’s task (*p* < 0.05). Mediating effect of BMI was found for the association between PACER laps and NoGo task, revealing a full mediator accounted for 81% of the total effect mediated (standardized indirect effect, –0.069, 95% CI –0.140, –0.020; standardized direct effect, 0.011, 95% CI –0.149, 0.165). No association was found for Attentional or Sternberg’s tasks. The findings suggest that school–aged adolescents with higher CRF level showed better planning and problem–solving abilities and cognitive flexibility. Additionally, the positive association of CRF with cognitive flexibility was sex–moderated and with inhibitory control was BMI–mediated.

## Introduction

Executive functions (EFs) refer to a set of cognitive processes responsible for maintaining behavior, emotion and thoughts (Diamond, 2013), developing from early childhood and through adolescence to adulthood (Romine and Reynolds, 2005). These functions can be categorized as the core EFs, which include inhibitory control (ability to control attention, behavior, thoughts, and/or emotions focusing on the goal and suppressing attention to other stimuli), working memory (retaining information in the mind and manipulating it), and cognitive flexibility (ability to change perspectives; i.e., to inhibit previous perspectives and activate into working memory a different perspective), and as higher–level EFs, which include planning and problem–solving abilities (Diamond, 2013). When combined, these functions enable us to evaluate actions ahead of time, resist the feeling to do something or react habitually, stay focused, elaborate ideas, reason, solve problems, adjust to changed demands or priorities, and see things from different points of view (Diamond and Ling, 2016). These are all crucial skills for success in various aspects of life, such as learning and academic achievements (Diamond and Ling, 2016). The relationship between EFs and academic skills such as reasoning, reading and arithmetic is already evident (Van der Sluis et al., 2007; Best et al., 2011). In addition to emotional and behavioral factors (Bidzan-Bluma and Lipowska, 2018), school activities demand an efficient EF performance as they are a model of an environment that requests autonomy and control of attentional functions, as well as organizing and planning (Cypel, 2006).

Approaches toward improving EFs are specially important during developmental stages of life such as adolescence, where neuroplasticity is heightened and the brain is more susceptible to changes from environmental factors (Blakemore, 2012). Cardiorespiratory fitness (CRF) is associated with the performance of EFs in adolescents (Huang et al., 2015; Pindus et al., 2015; Chen et al., 2017; Westfall et al., 2018), which may be partially explained by a greater efficiency in communication between different brain regions in those individuals with higher CRF (Opel et al., 2019; Peven et al., 2019). In young women, CRF is positively associated with better attention, working memory, planning and problem solving ability (Scott et al., 2016). In adolescents, higher CRF is related to improved inhibitory control, cognitive flexibility, and attention subdomain performance (Huang et al., 2015; Pindus et al., 2015; Westfall et al., 2018). Despite this, other authors did not observe any associations between CRF and working memory, cognitive flexibility and inhibitory control subdomain performance in adolescents (Stroth et al., 2009; Hogan et al., 2013; Tee et al., 2018). This discrepancy between those findings within the adolescent population may be due to the fact that this period is accompanied by several physical and hormonal changes, including increases in brain maturation influenced by sex hormones (Lenroot et al., 2007), this leads to change in cognitive ability, accuracy, reaction time, and reasoning from late adolescence until early adulthood (Gur and Gur, 2016). Indeed, there is still a need to establish evidence on the associations between CRF and the multiple EF domains, especially planning and problem–solving abilities, which are considered high–level cognitive functions. Therefore, this study aimed to investigate the association between CRF and performance in multiple EF domains in school–aged adolescents. It was hypothesized that adolescents with higher CRF would have greater performance in multiple EF domains.

## Materials and Methods

### Study Design

This cross–sectional study was conducted in the city of Natal, Brazil. We designed this study based on the Strengthening the Reporting of Observational Studies in Epidemiology (STROBE) guidelines (von Elm et al., 2007), while also following the ethical precepts of research in compliance with the Declaration of Helsinki, as well as the Resolution 466/2012 of the National Health Council of Brazil, with approval of the Institutional Ethics Committee (protocol no. 2.198.883).

### Participants

We recruited participants and collected data from September to December 2017. We used a convenience sample for this study, which consisted of students enrolled in middle school (years 6 – 9) of a private school. We used the following criteria for eligibility: boys and girls aged 11 – 16 years; consent and assent forms properly filled and signed, with consent from parents. The exclusion criteria were: physical or intellectual disability and/or clinical, neuromotor, psychological and/or cognitive contraindications (verified through the school’s pedagogical crew); not performing CRF assessment; not performing at least one cognitive test.

### Procedures

All participants underwent a clinical examination where body weight, height, pubertal stage, and daytime sleepiness were measured. Body mass index (BMI) was calculated using the ratio between weight and height squared (kg/m^2^). The BMI *z*-score of each participant was classified according to age and sex as “severe thinness, “thinness,” “healthy weight,” “overweight,” “obesity,” and “severe obesity” (WHO Multicentre Growth Reference Study Group, 2006). Pubertal stage was assessed through the peak height velocity (PHV). The PHV of each participant was classified as “pre–pubescent,” “pubescent,” or “post–pubescent” (Mirwald et al., 2002). Daytime sleepiness was assessed through the Pediatric Daytime Sleepiness Scale (Felden et al., 2016).

### PACER Test

CRF was evaluated through the progressive aerobic cardiovascular endurance run (PACER) test (Léger and Lambert, 1982). This test was performed in the school’s sports court in the morning (8:00–12:00 a.m.). The protocol consisted of individual sprints of 20 meters in round trips, during which velocity was controlled through the audio from a metronome. The starting speed was 8.5 km/h, followed by an increase of 0.5 km/h every 1 min. The test was carried out until voluntary exhaustion or if the participant was unable to maintain the rhythm established by the metronome, being behind the delimitated 20–meter line for two consecutive times. Participants were encouraged verbally to keep exercising for the longest time possible. The test was applied to groups of five children per turn, with there being one evaluator for each participant. The number of laps (main outcome), total distance (m), and maximum velocity (km/h) were registered at the end of the test. The number of laps reached by each participant was classified as being in a “healthy fitness zone” or “needs improvement,” according to sex and age (Cooper Institute for Aerobics Research, 2007).

### Cognitive Tasks

The cognitive tasks were applied using the Psychology Experiment Building Language (PEBL) version 2.0.4 on desktop computers (Mueller and Piper, 2014). Box 1 and Supplementary Data 1 show the cognitive tasks used to evaluate the EF domains. The tests were performed in the school’s computer laboratory during the morning (7:30–10:00 a.m.), without any environmental distractions. The tests were divided and performed on two distinct days to avoid mental fatigue by a team of trained researchers. On the first day, the following tests were performed: Berg’s Card Sorting task (BCST), Go/No–Go oddball task (GNG) and Sternberg’s Working Memory Search task (SMS). On the second day were administered: The Attentional Network task (ANT) and the Tower of London task (TOL). The tests were also divided in such a way that the duration of the tests was equivalent on both days. There was a minimum interval of 48 h between tests. In addition, the participants performed a familiarization session with the cognitive tests before the main assessment, respecting the same sequence and interval. Participants performed the tests after listening to a set of instructions read by the research team, following a script. The tests were self-administered, following the instructions on the computer screen. Participants performed the tests uninterruptedly unless the researchers noticed that a participant was diverting from the task instructions or if there were any questions about the test. If this was the case, participants were instructed to raise their hands to avoid drawing attention from their colleagues.

**Box 1**

Cognitive tasks used to evaluate the domains of executive functions.

**Table:** 

Cognitive tasks	Outcomes	Executive functions domains
TOL: Tower of London – task A	– Excess moves, s – Planning time, s – Solution time, s	Planning and problem–solving abilities
BCST: Berg’s Card Sorting task	– Completed categories – Perseverative errors	Cognitive flexibility
GNG: Go/No–Go oddball task	– Accuracy,% correct – Reaction time, s	Inhibitory control
SMS: Sternberg’s Working Memory Search task	– Accuracy,% correct – Reaction time, ms – Throughput	Working memory
ANT: Attentional Network task	– Accuracy,% correct – Reaction time, ms – Alerting, ms – Orienting, ms – Conflict, ms	Sustained attention

### Statistical Analysis

Data normality was verified through the Kolmogorov–Smirnov test and normal Q–Q plot. Continuous data were presented as median and interquartile range, while categorical data was presented as absolute and relative frequency. The generalized linear model (GzLM) or Fischer’s exact test were used for the bivariate analysis, when appropriate. We used the GzLM with maximum likelihood estimation to calculate the coefficient estimates (β) and its 95% Wald confidence interval (CI) for cognitive tasks using PACER laps as a predictor, controlling for covariates that were associated with cognitive outcomes in the bivariate analyses (*p* < 0.10). The following covariates were tested: age, sex, school year, pubertal stage, BMI categories, and daytime sleepiness (see Supplementary Tables 1, 2). The permanence of the covariate in the adjusted model followed the Wald test, absence of multicollinearity, as well as its ability to improve the model through the goodness of fit. The Omnibus test and Akaike information criterion (AIC) were used to assess the goodness of fit for the models. Multicollinearity between covariates was tested by bivariate analysis (GzLM). Multicollinearity was detected between the covariates age, school year, and pubertal stage. In the case of the models that should include these covariates, a model was tested for each covariate separately, as shown in Supplementary Table 3. The model that showed the better AIC was used in adjusted models presented in the main results. Gamma or linear distribution with an identity link was used in all models based on the goodness of fit. Afterward, the GzLM was used to determine the moderating effect of sex on the relationship between PACER laps and cognitive tasks. For this, added the term of interaction between PACER laps and sex. When an existing moderator effect was determined, meaning if an interaction was statistically significant (*p* < 0.05), the simple regression lines for each sex were inspected.

The mediating effect of BMI categories, school year, and daytime sleepiness on the relationship between PACER laps and cognitive tasks was tested by the mediation analysis using structural equation modeling (SEM). We used maximum Likelihood estimation and 5.000 bias-corrected (BC) bootstrap samples (95% BC CI level) for the total, direct and indirect effects. The effects and path coefficients were expressed as standardized estimates. The total effect of an independent variable on a dependent variable is decomposed into a direct and indirect effect. The indirect effect goes through a mediator variable (a and b paths in Figure 3), and the remaining effect reflects the direct effect (c’ path in Figure 3) (Rijnhart et al., 2017). Multivariate normality was assessed by the critical ratio (<5). Chi-square statistic (χ^2^) and related *p*-value, root mean square error of approximation (RMSEA), comparative fit index (CFI), and Tucker–Lewis index (TLI) were used as goodness of fit indices with cutoffs of *p*-value > 0.05 for χ^2^, ≤0.06 for RMSEA, and ≥0.95 for CFI and TLI (Hu and Bentler, 1999). The proportion (%) of the total effect mediated was calculated using the formula: 1 – (direct effect/total effect). An alpha level of *p* < 0.05 was used to determine statistical significance. All statistical procedures were performed using IBM SPSS Statistics and Amos for Win/v.25.0 (IBM Corp., Armonk, NY, United States).

## Results

### Sample Characteristics

**FIGURE 1:**
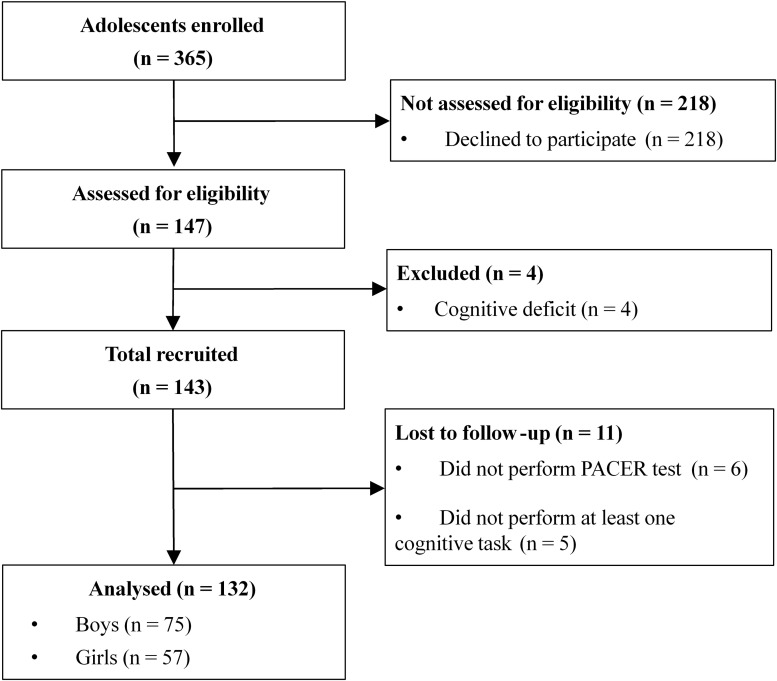
Study flow diagram. PACER, progressive aerobic cardiovascular endurance run test.

A total of 143 adolescents were eligible to participate in the study. However, 11 participants were excluded during the study due to not performing the CRF test (*n* = 6) or not performing at least one cognitive task (*n* = 5). Finally, a total of 132 participants were included in the data analysis (Figure 1). Table 1 shows the characteristics of the participants included in the study. Most participants were pubescent (60.6%) and were in a healthy fitness zone (72.7%).

**TABLE 1:** Sample characteristics of adolescents (*n* = 132).

	Overall	Boys	Girls	*P*^1^
***N* (%)**	132	75 (56.8)	57 (43.2)	
Age, years	12.0 (2.0)	12.0 (2.0)	12.0 (1.0)	0.561
**School year, *n* (%)**				
6th year	34 (25.8)	26 (34.7)^a^	8 (14.0)^b^	0.001
7th year	44 (33.3)	22 (29.3)^a^	22 (38.6)^a^	
8th year	29 (22.0)	9 (12.0)^a^	20 (35.1)^b^	
9th year	25 (18.9)	18 (24.0)^a^	7 (12.3)^a^	
**Pubertal stage, *n* (%)**				
Pre–pubescent	39 (29.5)	39 (52.0)^a^	0 (0.0)^b^	<0.001
Pubescent	80 (60.6)	32 (42.7)^a^	48 (84.2)^b^	
Post–pubescent	13 (9.8)	4 (5.3)^a^	9 (15.8)^b^	
Height, m	1.58 (0.12)	1.56 (0.2)	1.58 (0.06)	0.321
Weight, kg	51.5 (16.1)	54.0 (17.0)	49.9 (15.6)	0.218
BMI, kg/m^2^	20.4 (4.6)	20.5 (4.5)	20.2 (5.1)	0.499
**BMI categories, *n* (%)**				
Healthy weight	76 (57.6)	41 (54.7)	35 (61.4)	0.106
Overweight	37 (28.0)	19 (25.3)	18 (31.6)	
Obese	19 (14.4)	15 (20.0)	4 (7.0)	
PACER, laps	38 (24)	43 (31)	30 (18)	<0.001
PACER, m	760 (480)	860 (620)	600 (360)	<0.001
Vmax in PACER, km/h	10.5 (1.5)	11.0 (1.5)	10.0 (1.3)	<0.001
**PACER categories, *n* (%)**				
Healthy fitness zone	96 (72.7)	54 (72.0)	42 (73.7)	0.847
Needs improvement	36 (27.3)	21 (28.0)	15 (26.3)	
Daytime sleepiness, score	16.0 (8.0)	15.0 (8.3)	17.0 (8.0)	0.024

*Values are expressed as median and interquartile range for continuous variables and absolute and relative frequency for categorical variables. Bold values indicate significance at p < 0.05. Within a row, groups marked with different superscript letters are significantly different based on z–test (p < 0.05). ^1^P–value of the difference between the groups through the generalized linear model (continuous variables), or Fisher’s exact test (categorical variables). BMI, body mass index; PACER, progressive aerobic cardiovascular endurance run test; Vmax, maximum velocity.*

Table 2 shows the cognitive task performance. The RT on the Go/No–Go and ANT tasks was longer in the girls than boys (ps < 0.05). There were no statistical differences between boys and girls for the remaining cognitive tasks (ps > 0.05), but we found a trend for significance in the perseverative errors in BCST and alerting in ANT (ps < 0.10).

**TABLE 2:** Cognitive tasks performance of adolescents.

	Overall	Boys	Girls	*P*^1^
**TOL**	*n* = 131	*n* = 74	*n* = 57	
Excess moves	16.0 (12.0)	16.0 (11.3)	16.0 (12.0)	0.448
Planning time, s	4636.5 (2411.5)	4738.1 (2421.5)	4368.8 (1849.4)	0.919
Solution time, s	14968.4 (5982.9)	15077.9 (7040.1)	14668.8 (5254.9)	0.707
**BCST**	*n* = 132	*n* = 75	*n* = 57	
Completed categories	6 (3.75)	6.0 (4.0)	6.0 (3.0)	0.695
Perseverative errors	16 (8.8)	15.0 (8.0)	17.0 (9.0)	**0.072**
**GNG**	*n* = 127	*n* = 72	*n* = 55	
Accuracy Go,% correct	98.4 (3.9)	99.0 (3.7)	97.7 (3.1)	0.640
RT Go, ms	411.5 (66.9)	401.0 (67.5)	429.2 (51.9)	**0.001**
	*n* = 125	*n* = 71	*n* = 54	
Accuracy NoGo,% correct	96.9 (6.3)	96.8 (9.4)	96.9 (6.3)	0.432
RT NoGo, ms	523.7 (81.6)	504.7 (60.7)	546.2 (82.9)	**<0.001**
**SMS**	*n* = 129	*n* = 73	*n* = 56	
Accuracy,% correct	92.0 (6.3)	92.0 (6.3)	92.0 (5.7)	0.443
RT, ms	813.8 (123.9)	818.9 (147.4)	808.9 (94.0)	0.459
Throughput	3.27 (0.61)	3.27 (0.63)	3.30 (0.57)	0.183
**ANT**	*n* = 132	*n* = 75	*n* = 57	
Accuracy,% correct	97.6 (4.5)	97.6 (4.5)	97.9 (5.4)	0.910
RT, ms	629.6 (167.4)	611.6 (150.4)	675.7 (158.0)	**0.002**
Alerting, ms	59.5 (68.5)	60.1 (70.2)	58.3 (68.6)	**0.060**
Orienting, ms	37.4 (44.7)	40.9 (45.9)	35.2 (45.2)	0.240
Confict, ms	89.4 (62.1)	87.2 (51.2)	91.3 (82.7)	0.272

*Values are expressed as median and interquartile range. Bold values indicate significance at p < 0.10. ^1^P–value of the difference between the groups through the generalized linear model. ANT, Attentional Network task; BCST, Berg’s Card Sorting task; GNG, Go/No–Go oddball task; RT, reaction time; SMS, Sternberg’s Working Memory Search task; TOL, Tower of London task.*

### Relationship Results

Table 3 shows the analysis using PACER laps as a continuous predictor of cognitive tasks. After adjusting for covariates (see covariates in Supplementary Table 1), we found that PACER laps were negatively related to planning and solution time in TOL and perseverative errors in BCST (*p* < 0.05). We also found a negative relationship between PACER laps and RT on the NoGo task, but the significance disappeared after adjusting for covariates. No relationships were found for SMS and ANT tasks (ps > 0.05).

**TABLE 3:** Generalized linear models of PACER laps as a predictor of cognitive tasks in adolescents.

	Unadjusted		Adjusted^1^	
	β (95% CI)	*P*	β (95% CI)	*P*
**TOL**	*n* = 131		*n* = 131	
Excess moves	−0.013(-0.082,0.057)	0.724	−0.013(-0.099,0.073)	0.766
Planning time, s	−18.5(-32.4,−4.7)	**0.009**	−17.1(-31.9,−2.3)	**0.023**
Solution time, s	−52.9(-85.9,−19.9)	**0.002**	−44.6(-75.1,−14.2)	**0.004**
**BCST**	*n* = 132		*n* = 132	
Completed categories	0.013(-0.006,0.032)	0.176	0.011(-0.009,0.031)	0.286
Perseverative errors	−0.087(-0.149,−0.024)	**0.007**	−0.073(-0.133,−0.013)	**0.017**
**GNG**	*n* = 127		*n* = 127	
Accuracy Go,% correct	0.011(-0.019,0.040)	0.479	*	
RT Go, ms	−0.237(-0.785,0.311)	0.397	0.489(-0.198,1.176)	0.163
	*n* = 125		*n* = 125	
Accuracy NoGo,% correct	0.013(-0.037,0.063)	0.616	0.002(-0.049,0.052)	0.948
RT NoGo, ms	−0.691(-1.193,−0.188)	**0.007**	0.039(-0.487,0.565)	0.883
**SMS**	*n* = 129		*n* = 129	
Accuracy,% correct	0.011(-0.030,0.052)	0.588	−0.004(-0.045,0.037)	0.844
RT, ms	−0.552(-1.402,0.297)	0.203	−0.401(-1.239,0.436)	0.348
Throughput	0.000(-0.004,0.004)	0.891	−0.001(-0.005,0.003)	0.694
**ANT**	*n* = 132		*n* = 132	
Accuracy,% correct	0.024(-0.051,0.098)	0.534	*	
RT, ms	−0.494(-1.666,0.678)	0.409	0.143(-0.840,1.126)	0.776
Alerting, ms	−0.392(-0.801,0.017)	0.060	−0.275(-0.734,0.184)	0.240
Orienting, ms	0.031(-0.303,0.365)	0.855	*	
Conflict, ms	−0.170(-0.607,0.267)	0.446	−0.116(-0.555,0.324)	0.605

Values are expressed as coefficient estimates (β) and 95% confidence interval (CI). All significant models presented a p < 0.05 in the Omnibus test. Bold values indicate significance at p < 0.05. ^1^Adjusted model for the covariates presented in Supplementary Table 1. *No covariate was associated with this outcome in the bivariate analysis. ANT, Attentional Network task; BCST, Berg’s Card Sorting task; GNG, Go/No–Go oddball task; PACER, progressive aerobic cardiovascular endurance run test; RT, reaction time; SMS, Sternberg’s Working Memory Search task; TOL, Tower of London task.

### Moderating Effects

**FIGURE 2:**
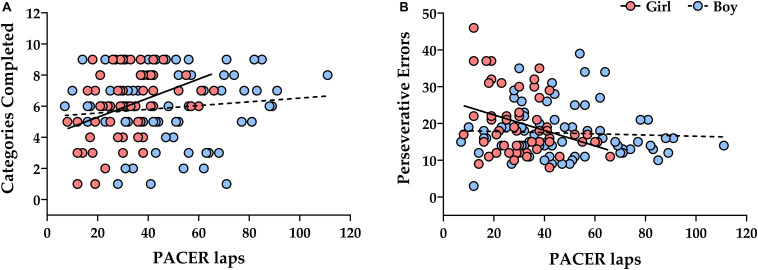
Linear association of PACER lap with categories completed (A) and perseverative errors (B) in Berg’s Card Sorting task in boys (*n* = 75) and girls (*n* = 57). Categories completed **(A)** – Boys: Generalized linear model, β = 0.007, *p* = 0.572. Girls: Generalized linear model, β = 0.056, *p* = 0.001. Perseverative errors **(B)** – Boys: Generalized linear model, β = –0.031, *p* = 0.291. Girls: Generalized linear model, β = –0.226, *p* = 0.005. PACER, progressive aerobic cardiovascular endurance run test.

Since sex differences were observed for some cognitive tasks and the PACER test, we analyzed the moderating effect of sex on the relationship between PACER laps and cognitive tasks. The relationship between PACER laps and BCST was sex–moderated, both for completed categories (PACER laps and sex interaction: β = –0.049, 95% CI –0.091, –0.008, *p* = 0.020) and for perseverative errors (PACER laps and sex interaction: β = 0.194, 95% CI 0.025, 0.363, *p* = 0.024). Figure 2 and Supplementary Table 4 shows that PACER laps were negatively related with completed categories and positively related to perseverative errors only in girls (*p* < 0.05). No moderating effect of sex was found for TOL, GNG, SMS, or ANT tasks (ps > 0.05).

### Mediating Effects

**FIGURE 3:**
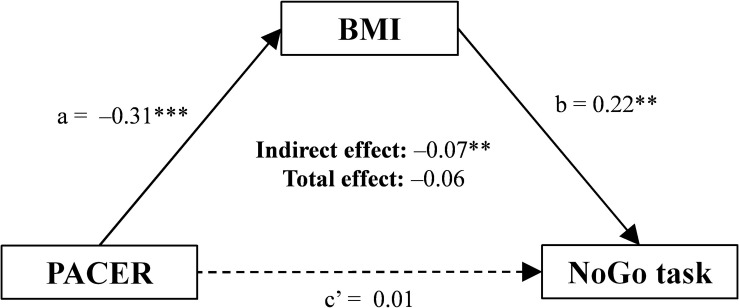
Mediating effect of BMI *z*-score categories on the relationship between PACER laps and NoGo task (ms) in adolescents, controlling for sex and school year (*n* = 125). Values are expressed as standardized estimates. Path a = effect of the PACER on the BMI; Path b = effect of the BMI on the NoGo task. Path c’ = direct effect of the PACER on the NoGo task, controlling for BMI. BMI, body mass index; PACER, progressive aerobic cardiovascular endurance run test. ^∗∗^*P* < 0.01. ^∗∗∗^*P* < 0.001.

We found that there were significant relationships between the PACER test and BMI categories, school year, and daytime sleepiness (*p* < 0.05). We also found a significant relationship between these covariates and some cognitive tests (Supplementary Table 2). Therefore, due to these strong relationships, we tested the mediating effects of BMI categories, school year, and daytime sleepiness. The BMI categories was a full mediator on the relationship between PACER laps and RT on the NoGo task, as observed by standardized indirect effect (–0.069, 95% CI –0.140, –0.020, *p* = 0.004), standardized direct effect (0.011, 95% CI –0.149, 0.165, *p* = 0.912) and standardized total effect (–0.057, 95% CI –0.221, 0.103, *p* = 0.503), controlling for sex and school year (Figure 3). The proportion of the total effect mediated of BMI on NoGo task estimated by PACER was 81%. This mediation model showed satisfactory fit indices, χ^2^/df = 1.331, p = 0.249, CFI = 0.995, TLI = 0.951, RMSEA = 0.052, 90% CI 0.000, 0.251. No mediating effect of BMI categories was found for TOL, BCST, or ANT tasks. No mediating effect of school year and daytime sleepiness was found for any cognitive tasks.

*Post hoc* statistical power analyses showed that all significant results reached a power (1–β) equal or higher than 82% based on sample size, achieved effect size, and an alpha of 5% (G^∗^Power, version 3.1.9.4; Institute for Experimental Psychology in Dusseldorf, Germany).

## Discussion

The main findings of this study were: (a) there were positive associations of CRF with planning and problem–solving abilities and cognitive flexibility in school–aged adolescents; (b) the association between CRF and cognitive flexibility was moderated by sex, reflecting in stronger association for girls; (c) the association between CRF and inhibitory control was fully mediated by BMI. To the best of our knowledge, this is the first study to investigate the association between CRF and multiple EF domains, including higher–level EFs (i.e., planning and problem–solving abilities) using well–established evaluations in a population of pubescent and post–pubescent adolescents. We also observed a moderating effect of sex and a mediating effect of BMI which allows us to contribute toward elucidating the role of sexual dimorphisms and nutritional status, respectively, in the association between CRF and cognitive performance in adolescents.

Good planning and problem–solving abilities indicate more refined superior order EFs, in which the individual’s brain requires less effort (i.e., pre–frontal cortex usage) in order to perform cognitive tasks efficiently. Our findings report that a higher CRF is associated with a better EF performance in adolescents. CRF is already recognized as a clinical marker of physical health for adolescents (World Health Organization [WHO], 2010), and this strengthens its connection with cognitive health (Álvarez-Bueno et al., 2017). The literature is scarce regarding the mechanisms that explain the association of CRF with EFs. Findings by Opel et al. (2019) indicate that greater CRF favors the structure of the white matter, which is responsible for communication between different brain regions and, consequently, contributes to the cognitive function. Peven et al. (2019) have found that CRF is associated with a better EF by suppressing connectivity between brain regions that are involved with sensory processing irrelevant to the task.

Regular physical activity, especially when performed at a moderate–to–vigorous intensity (e.g., sports and aerobic exercises), leads to increased CRF (Costigan et al., 2016; Minatto et al., 2016) and, consequently, cognitive capacity (Álvarez-Bueno et al., 2017) in adolescents. Still, physical activity is an environmental factor that increases neuroplasticity, inducing important structural and functional changes in the brain. These changes include increased cerebral blood flow, increased gray matter volume in the frontal and hippocampal regions, and higher blood levels of brain–derived neurotrophic factor (Mandolesi et al., 2018). Thus, adolescents who regularly engage in physical activity can obtain benefits toward their cardiovascular and cerebrovascular health, which translates into an improved cognitive capacity. However, individual factors may also modulate their CRF response to environmental stimuli. For example, genetic factors determine 47% of the change in CRF after an exercise training program (Bouchard et al., 1999). Adolescents who have greater physical fitness and/or EFs performance at baseline seem to benefit less from physical exercise programs (Strong et al., 2005; Moreau et al., 2017). Genetic and environmental factors can increase or limit the effect of physical exercise on CRF and EFs.

We did not find any independent associations between CRF and working memory, attention or inhibitory control. The direct effects of CRF and inhibitory control were not seen after adjusting for BMI. For this reason, we found that BMI is a mediator of the association between CRF and inhibitory control. Studies have shown that excess body weight is not only associated with a lower CRF in adolescents (Ortega et al., 2007), but also negatively and independently impacts EFs (Tee et al., 2018). Although these mechanisms are poorly understood, it is believed that the state of low-grade inflammation generated by body fat can trigger neuroinflammatory and neuroplastic processes that modify brain connectivity (Samara et al., 2020). These changes hinder communication between the inhibitory pre-frontal regions of the brain and its reward circuits, which may lead to predominantly hedonistic mechanisms, impairing the regulation of food intake and sedentary behavior (Gupta et al., 2015; Samara et al., 2020). There is conflicting literature in regards to the relationship between CRF and inhibitory control (Stroth et al., 2009; Huang et al., 2015; Pindus et al., 2015; Tee et al., 2018; Westfall et al., 2018). The mediating effect of BMI, along with differences in the prevalence of excess body weight, may help to explain these disparities. For example, the prevalence of excess body weight in our sample was 42% (considering overweight + obese), while another study that did not find associations showed a prevalence of 33% (Tee et al., 2018). These prevalence rates are two to three times higher when compared to studies that found positive associations [i.e., Huang et al. (2015) = 14%; Pindus et al. (2015) = 17%]. Thus, we observed that the high prevalence of overweightness in our study might have inferred in the absence of a direct effect of the association between CRF and inhibitory control after adjusting for BMI, which did not happen in other studies in adolescents with a low prevalence of excess body weight.

We found that CRF is positively associated with cognitive flexibility performance in adolescents, with sex having a moderating effect. This finding showed that the associations are stronger in girls. This may have occurred due to the differences in pubertal maturation between boys and girls of the same age, although there was no moderating or meditating effect of the pubertal stage, age or BMI. Girls begin puberty one or two years earlier than boys, and the high exposure to sexual hormones influences cerebral organization and development (Neufang et al., 2009; Blakemore, 2012). It should be noted that all participants in the girls’ group were either pubescent or post–pubescent (84.2 and 15.8%, respectively), while a large part of the boys’ group was pre–pubescent (52%). The girls probably already showed a more advanced process of frontal region maturation when compared to boys. Sex hormones influence the maturation of the front lobes (Herting et al., 2014), which are related to EFs, as well as being the region in the brain which takes the longest time to mature (Gogtay et al., 2004). Thus, we presumed that girls had a more advanced cerebral maturation when compared to boys, especially in the frontal region. The higher levels of estradiol hormone may have influenced maturation of brain areas related to the cortical region, which are related to cognitive flexibility. These factors may have modified the interaction of CRF with cognitive flexibility between sexes.

Finally, adolescence is the period in life in which neuroplasticity is at its highest, making the brain more susceptible to changes from environmental factors such as physical activity/exercise practice and offering more possibilities to stimulate cognitive function (Blakemore, 2012). Paradoxically, it is also the period with the highest prevalence of physical inactivity (Kohl et al., 2012). Current literature already supplies a strong subsidy for creating strategies to promote behavioral changes in children and adolescents, like increasing physical activity and decreasing sitting time (World Health Organization [WHO], 2010; Tremblay et al., 2017). School may be a determining environment to enable strategies toward behavior change since it is a place in which children and adolescents spend a large time of their day, as well as being an environment in which several behavioral habits that can be perpetuated throughout adulthood are created.

Regarding the strengths of the present study, we highlight the investigation of multiple domains of EFs, including planning and problem-solving abilities, in a sample of school-aged adolescents. Also, we tested structural equation models (SEM), which revealed the moderating effect of sex and the mediating effect of BMI on the association between CRF with cognitive flexibility and inhibitory control, respectively. Some limitations of our study should be mentioned. First, we assessed CRF indirectly through the PACER test. Nevertheless, the PACER test is an internationally recognized and recommended test to estimate the aerobic capacity of adolescents, as well as being a test that is more specific to the manners in which adolescents usually perform physical activity in school and non–school environments. This may somehow favor the participants to reach their maximum aerobic capacity, as opposed to what could occur on a test performed in a laboratory setting using a treadmill or cycle ergometer. Second, the pubertal stage was not homogeneous between boys and girls. Only 42.7% of boys were classified as pubescent, for example, while the same was true for 84.2% of girls. This may have happened due to the sample being chosen by convenience. Third, this is a cross-sectional study, which does not allow establishing a cause-effect relationship between CRF and EFs. In addition, it does not exclude an inverse causality (i.e., EFs modulating CRF). We believe it is unlikely that greater EF can induce an improvement in CRF. However, we cannot rule out the possibility that this relationship is created by adolescents who are oriented toward success and who are more likely to achieve greater performance in the activities performed, whether in cognitive or physical fitness tasks (Thøgersen-Ntoumani and Ntoumanis, 2006).

## Conclusion

The findings of our study suggest that CRF is associated with planning and problem–solving abilities and cognitive flexibility performance in school–aged adolescents. Moreover, sex moderated the effect of the association on the cognitive flexibility outcomes, revealing that higher levels of CRF were more strongly associated with better cognitive flexibility performance in girls. The association between CRF and inhibitory control was fully mediated by the BMI in boys and girls. Despite this, there were no associations between CRF and the other EF subdomains (e.g., working memory or sustained attention) in boys and girls.

## Data Availability Statement

The raw data supporting the conclusions of this article will be made available by the authors, without undue reservation.

## Ethics Statement

The studies involving human participants were reviewed and approved by the Research Ethics Committee of the Federal University of Rio Grande do Norte. Written informed consent to participate in this study was provided by the participants’ legal guardian/next of kin.

## Author Contributions

LC, RB, EC, and JB: conceptualization. LC and GS: data curation. RB and YF: formal analysis. LC, RB, DS, GS, MD, FL, and LF-J: investigation. LC, RB, and YF: methodology. LC: project administration. EC and JB: resources and supervision. LC, RB, YF, and DS: writing – original draft. GS, MD, FL, LF-J, EC, and JB: writing – review and editing. All authors have read and agreed to the published version of the manuscript.

## Conflict of Interest

The authors declare that the research was conducted in the absence of any commercial or financial relationships that could be construed as a potential conflict of interest.
