# Supplementary material for: Cardiorespiratory Fitness and Performance in Multiple Domains of Executive Functions in School–Aged Adolescents
Source: Front Physiol. 2021 Mar 2;12:640765. doi: 10.3389/fphys.2021.640765 (PMC7960783; doi:10.3389/fphys.2021.640765)
Supplement: Supplementary file 1 [file Table_1.DOCX]

Supplementary Data 1 | Cognitive tasks used to evaluate executive functions.

Tower of London

The Tower of London (TOL) task was used to assess planning and problem-solving abilities. We used task A, which consists of eight problems with five disks with increasing difficulties. During the task, the participants had to move colored disks from their initial position to a new pre-determined formation, searching for the most efficient solution with the fewest number of movements to reach their goal. The main dependent variable was the total number of extra movements throughout the eight tasks (amount of movements performed minus 51, the minimum number needed to solve the problems). However, total time spent in the test was also recorded (time needed to reach the final goal), as well as planning time (time which the participant spent planning their answers, meaning the time between presenting the problem and the movement of the first disk) (Phillips et al., 1999).

Berg’s Card Sorting task

Berg’s Card Sorting task (BCST) was used to assess cognitive flexibility. It is a task-based on trial and error, in which the participant needed to classify 128 cards, one card at a time, according to an unknown rule (number, color, or form). The rule changed regularly during the task, requesting participants to modify their strategy and discover the new rule as quickly as possible. Four key-cards were shown in the upper part of the screen as a guide to help determine in which of the four stacks the card should be classified. Feedback was given after each sorted card, (correct or incorrect), and the rule changed after 10 right answers (corresponding to one finished category). The test continued until the participant classified all 128 cards, or successfully completed nine categories (whichever happened first). The main results of the BCST were the number of categories completed and the number of perseverative errors (incorrect answers according to the classification rule in progress, from the classification rule of the previous block) (Fox et al., 2013).

Go/No-Go Oddball

The Go/No-Go Oddball (GNG) task was used to assess inhibitory control. It is a task of inhibiting answers, where one motor answer was to be executed or inhibited. During the task, participants watched a sequence of letters being presented and answered pressing the mouse button. The presentation was a 2 × 2 matrix with four stars (one in each quadrant). A single letter (P or R) was presented in each quadrant for 500 ms, with an inter-stimuli interval of 1500 ms. In the first condition (P = Go), the students had to press the button in answer to the target-letter P and inhibit their answer to the non-target letter R. The ratio of target to non-target letters was 80:20. The second condition as opposed to the first (R = Go), in which the participants had to press the button in answering the target letter R and inhibit an answer to the non-target letter P. The number of attempts was 160 in both conditions, adding up to a total of 320. The measures included in the task results were accuracy (percentage of right answers) and reaction time (from answers to decisions). Excessively short (< 150 ms) or long (> 20 s) times were excluded before calculating the meantime for each participant (Bezdjian et al., 2009).

Sternberg’s Working Memory Search task

Sternberg’s Working Memory Search (SMS) task was used to assess working memory. The task involved a series of neural subsystems, in which the objective was to search for the information in the extension of immediate memory. Furthermore, the latency of the answers (time from the start of the stimulus until the answer) revealed the time that the subject spent recovering the information. The test requested that the participant memorized a set of consonants (two, four, or six – changing the difficulty level) and pointed if the letters which were shown afterward were absent or present in the set of consonants they memorized. Therefore, we also examined throughput in addition to accuracy and reaction time, which is a return rate of the maximum capacity of working memory, calculated as follows: throughput = [(accuracy ˗ 0.5) × 2 × number of letters] (Sternberg, 1966).

Attentional Network task

The Attentional Network Task (ANT) was used to assess three distinct components of sustained attention (i.e., alerting, orienting, and conflict). This task consisted of four blocks. The first block intended to familiarize the subject with the test and three target conditions (congruent, incongruous, and neutral), for minutes. The next three blocks lasted five minutes each, referring to the text itself, whose answers were counted in the score. The ANT requested that participants focus their attention on the arrow in the central position while shifting attention to the arrows in the other positions, in order to inhibit irrelevant stimuli. The stimuli consisted of a row of five horizontal black lines presented visually, with arrowheads pointing left or right, on a gray background. The measured results were based on the precision of correct answers (accuracy), reaction time (ms) and awareness indexes (perception of if there is something to be answered), orientation (directing or diverting attention to a determined location), and conflict (suppressing irrelevant information to focus on the request which had to be answered) (Fan et al., 2005).

REFERENCES

Bezdjian, S., Baker, L. A., Lozano, D. I., and Raine, A. (2009). Assessing inattention and impulsivity in children during the Go/NoGo task. *Br. J. Dev. Psychol.* 27, 365–383. doi:10.1348/026151008X314919.

Fan, J., McCandliss, B. D., Fossella, J., Flombaum, J. I., and Posner, M. I. (2005). The activation of attentional networks. *Neuroimage* 26, 471–9. doi:10.1016/j.neuroimage.2005.02.004.

Fox, C. J., Mueller, S. T., Gray, H. M., Raber, J., and Piper, B. J. (2013). Evaluation of a short-form of the Berg Card Sorting Test. *PLoS One* 8, e63885. doi:10.1371/journal.pone.0063885.

Phillips, L. H., Wynn, V., Gilhooly, K. J., Della Sala, S., and Logie, R. H. (1999). The role of memory in the Tower of London task. *Memory* 7, 209–31. doi:10.1080/741944066.

Sternberg, S. (1966). High-speed scanning in human memory. *Science* 153, 652–4. doi:10.1126/science.153.3736.652.
