# Supplementary material for: Cardiorespiratory Fitness and Performance in Multiple Domains of Executive Functions in School–Aged Adolescents
Source: Front Physiol. 2021 Mar 2;12:640765. doi: 10.3389/fphys.2021.640765 (PMC7960783; doi:10.3389/fphys.2021.640765)
Supplement: Supplementary file 2 [file Table_2.DOCX]

**Supplementary Table 1 |** Covariates included in the adjusted models in Table 3.

|  | **Covariates** |
| --- | --- |
| **TOL** |  |
| Excess moves | BMI |
| Planning time, s | School year |
| Solution time, s | School year |
| **BCST** |  |
| Completed categories | School year |
| Perseverative errors | Sex |
| **GNG** |  |
| Accuracy Go, % correct | None |
| RT Go, ms | School year, Sex, BMI |
| Accuracy NoGo, % correct | School year, Sleepiness |
| RT NoGo, ms | School year, Sex, BMI |
| **SMS** |  |
| Accuracy, % correct | School year, Sleepiness |
| RT, ms | Sleepiness |
| Throughput | School year, Sleepiness |
| **ANT** |  |
| Accuracy, % correct | None |
| RT, ms | Sex, Sleepiness |
| Alerting, ms | Sex |
| Orienting, ms | None |
| Conflict, ms | Age |

ANT, Attentional Network task; BCST, Berg’s Card Sorting task; BMI, body mass index; GNG, Go/No-Go oddball task; PACER, progressive aerobic cardiovascular endurance run test; RT, reaction time; SMS, Sternberg’s Working Memory Search task; TOL, Tower of London task.
