# Supplementary material for: Cardiorespiratory Fitness and Performance in Multiple Domains of Executive Functions in School–Aged Adolescents
Source: Front Physiol. 2021 Mar 2;12:640765. doi: 10.3389/fphys.2021.640765 (PMC7960783; doi:10.3389/fphys.2021.640765)
Supplement: Supplementary file 3 [file Table_3.DOCX]

**Supplementary Table 2 |** Bivariate analysis among cognitive tasks and covariates using the generalized linear model.

|  | **Age** | | **School year** | | **Puberty** | | **Sex** | | **BMI** | | | **Sleepiness** | |
| --- | --- | --- | --- | --- | --- | --- | --- | --- | --- | --- | --- | --- | --- |
|  | **Wald ꭓ^2^** | **P** | **Wald ꭓ^2^** | **P** | **Wald ꭓ^2^** | **P** | **Wald ꭓ^2^** | **P** | | **Wald ꭓ^2^** | **P** | **Wald ꭓ^2^** | **P** |
| **TOL** |  |  |  |  |  |  |  |  | |  |  |  |  |
| Excess moves | 0.02 | 0.897 | 1.78 | 0.618 | 0.27 | 0.873 | 0.58 | 0.448 | | 7.65 | **0.022** | 1.10 | 0.295 |
| Planning time, s | 2.33 | 0.127 | 8.78 | **0.032** | 1.24 | 0.537 | 0.01 | 0.919 | | 0.28 | 0.868 | 1.14 | 0.285 |
| Solution time, s | 13.21 | **<0.001** | 21.58 | **<0.001** | 6.71 | **0.035** | 0.13 | 0.722 | | 1.01 | 0.602 | 0.41 | 0.523 |
| **BCST** |  |  |  |  |  |  |  |  | |  |  |  |  |
| Completed categories | 0.75 | 0.387 | 14.64 | **0.002** | 3.70 | 0.157 | 0.15 | 0.695 | | 0.69 | 0.707 | 0.86 | 0.353 |
| Perseverative errors | 0.00 | 0.991 | 5.22 | 0.156 | 1.17 | 0.558 | 3.24 | **0.072** | | 0.67 | 0.714 | 1.16 | 0.282 |
| **GNG** |  |  |  |  |  |  |  |  | |  |  |  |  |
| Accuracy Go, % correct | 0.19 | 0.665 | 2.28 | 0.516 | 0.13 | 0.937 | 0.22 | 0.640 | | 1.47 | 0.479 | 0.37 | 0.540 |
| RT Go, ms | 0.34 | 0.557 | 7.77 | **0.051** | 1.17 | 0.556 | 10.85 | **0.001** | | 5.48 | **0.065** | 0.25 | 0.618 |
| Accuracy NoGo, % correct | 6.46 | **0.011** | 18.09 | **<0.001** | 4.99 | **0.083** | 0.62 | 0.432 | | 2.11 | 0.348 | 3.56 | **0.059** |
| RT NoGo, ms | 4.89 | **0.027** | 15.66 | **0.001** | 1.03 | 0.599 | 17.35 | **<0.001** | | 4.76 | **0.093** | 3.21 | **0.074** |
| **SMS** |  |  |  |  |  |  |  |  | |  |  |  |  |
| Accuracy, % correct | 13.60 | **<0.001** | 22.78 | **<0.001** | 15.62 | **<0.001** | 0.59 | 0.443 | | 1.01 | 0.603 | 9.66 | **0.002** |
| RT, ms | 2.93 | **0.087** | 4.36 | 0.225 | 0.53 | 0.767 | 0.55 | 0.459 | | 2.83 | 0.243 | 5.34 | **0.021** |
| Throughput | 13.24 | **<0.001** | 18.42 | **<0.001** | 17.68 | **<0.001** | 1.77 | 0.183 | | 2.26 | 0.324 | 7.02 | **0.008** |
| **ANT** |  |  |  |  |  |  |  |  | |  |  |  |  |
| Accuracy, % correct | 0.45 | 0.504 | 0.943 | 0.815 | 0.78 | 0.678 | 0.01 | 0.910 | | 3.56 | 0.168 | 1.28 | 0.257 |
| RT, ms | 1.89 | 0.169 | 3.46 | 0.326 | 2.72 | 0.321 | 9.29 | **0.002** | | 1.11 | 0.574 | 3.78 | **0.052** |
| Alerting, ms | 0.05 | 0.820 | 1.38 | 0.709 | 0.00 | 0.999 | 3.54 | **0.060** | | 0.07 | 0.963 | 0.97 | 0.325 |
| Orienting, ms | 0.14 | 0.703 | 2.42 | 0.490 | 3.59 | 0.166 | 1.38 | 0.240 | | 0.31 | 0.857 | 0.43 | 0.512 |
| Conflict, ms | 4.02 | **0.045** | 4.43 | 0.218 | 1.83 | 0.400 | 1.21 | 0.272 | | 2.06 | 0.357 | 0.02 | 0.893 |

Bold values indicate significance at p < 0.10.

ANT, Attentional Network task; BCST, Berg’s Card Sorting task; BMI, body mass index categories; GNG, Go/No-Go oddball task; PACER, progressive aerobic cardiovascular endurance run test; RT, reaction time; SMS, Sternberg’s Working Memory Search task; TOL, Tower of London task.
