# Supplementary material for: Cardiorespiratory Fitness and Performance in Multiple Domains of Executive Functions in School–Aged Adolescents
Source: Front Physiol. 2021 Mar 2;12:640765. doi: 10.3389/fphys.2021.640765 (PMC7960783; doi:10.3389/fphys.2021.640765)
Supplement: Supplementary file 4 [file Table_4.DOCX]

**Supplementary Table 3 |** Comparison of the adjusted models that showed multicollinearity between covariates.

|  | **Covariates** | **β (95% CI)** | **P** | **Omnibus test** | **AIC** |
| --- | --- | --- | --- | --- | --- |
| **TOL** |  |  |  |  |  |
| Solution time, s |  |  |  |  |  |
| Model 1 | None | –52.9 (–85.9, –19.9) | 0.002 |  |  |
| Model 2 | School year | –44.6 (–75.1, –14.2) | 0.004 | **<0.001** | **2570.8** |
| Model 3 | Age | –41.8 (–73.3, –10.2) | 0.010 | <0.001 | 2576.5 |
| Model 4 | Pubertal stage | –62.0 (–91.6, –32.3) | <0.001 | 0.001 | 2582.6 |
| **GNG** |  |  |  |  |  |
| Accuracy NoGo, % correct |  |  |  |  |  |
| Model 1 | None | 0.013 (–0.037, 0.063) | 0.616 |  |  |
| Model 2 | School year, Sleepiness | 0.002 (–0.049, 0.052) | 0.948 | **0.009** | **792.4** |
| Model 3 | Age, Sleepiness | –0.005 (–0.057, 0.047) | 0.852 | 0.026 | 794.4 |
| Model 4 | Pubertal stage, Sleepiness | 0.011 (–0.040, 0.061) | 0.679 | 0.102 | 797.9 |
| RT NoGo, ms |  |  |  |  |  |
| Model 1 | None | –0.691 (–1.193, –0.188) | 0.007 |  |  |
| Model 2 | School year, Sex, BMI | 0.039 (–0.487, 0.565) | 0.883 | **<0.001** | **1359.9** |
| Model 3 | Age, Sex, BMI | –0.030 (–0.564, 0.505) | 0.914 | <0.001 | 1361.6 |
| **SMS** |  |  |  |  |  |
| Accuracy, % correct |  |  |  |  |  |
| Model 1 | None | 0.011 (–0.030, 0.052) | 0.588 |  |  |
| Model 2 | School year, Sleepiness | –0.004 (–0.045, 0.037) | 0.844 | **<0.001** | **776.5** |
| Model 3 | Age, Sleepiness | –0.008 (–0.047, 0.031) | 0.701 | <0.001 | 778.3 |
| Model 4 | Pubertal stage, Sleepiness | 0.011 (–0.026, 0.048) | 0.572 | <0.001 | 778.3 |
| Throughput |  |  |  |  |  |
| Model 1 | None | 0.000 (–0.004, 0.004) | 0.891 |  |  |
| Model 2 | School year, Sleepiness | –0.001 (–0.005, 0.003) | 0.694 | **0.001** | **187.9** |
| Model 3 | Age, Sleepiness | –0.001 (–0.005, 0.003) | 0.613 | 0.004 | 191.2 |
| Model 4 | Pubertal stage, Sleepiness | 0.001 (–0.003, 0.005) | 0.599 | 0.011 | 193.4 |

Values are expressed as coefficient estimates (β) and 95% confidence interval (CI).

Bold values indicate the better model adjusted based on the AIC and Omnibus test.

AIC, Akaike information criterion; GNG, Go/No–Go oddball task; SMS, Sternberg’s Working Memory Search task; TOL, Tower of London task.
