# Supplementary material for: Cardiorespiratory Fitness and Performance in Multiple Domains of Executive Functions in School–Aged Adolescents
Source: Front Physiol. 2021 Mar 2;12:640765. doi: 10.3389/fphys.2021.640765 (PMC7960783; doi:10.3389/fphys.2021.640765)
Supplement: Supplementary file 5 [file Table_5.DOCX]

**Supplementary Table 4 |** Generalized linear models with PACER laps as a predictor of the Berg’s Card Sorting task in boys (n = 75) and girls (n = 57).

|  | **Unadjusted** |  | **Adjusted** |  |  |
| --- | --- | --- | --- | --- | --- |
|  | **β (95% CI)** | **P** | **β (95% CI)** | **P** | **Covariate** |
| **Completed categories** |  |  |  |  |  |
| Boys | 0.007 (–0.017, 0.030) | 0.572 | –0.001 (–0.025, 0.022) | 0.908 | School year |
| Girls | 0.056 (0.021, 0.090) | **0.001** | 0.048 (0.005, 0.090) | **0.027** | BMI |
| **Perseverative errors** |  |  |  |  |  |
| Boys | –0.031 (–0.089, 0.027) | 0.291 | * |  |  |
| Girls | –0.226 (–0.384, –0.067) | **0.005** | –0.205 (–0.363, –0.047) | **0.011** | School year |

Values are expressed as coefficient estimates (β) and 95% confidence interval (CI).

Bold values indicate significance at p < 0.05.

All significant models presented a p < 0.05 in the Omnibus test.

* No covariate was associated with this outcome in the bivariate analysis.

BMI, body mass index; PACER, progressive aerobic cardiovascular endurance run test.
